# Supplementary material for: Platinized graphene fiber electrodes uncover direct spleen-vagus communication
Source: Commun Biol. 2021 Sep 17;4:1097. doi: 10.1038/s42003-021-02628-7 (PMC8448843; doi:10.1038/s42003-021-02628-7)
Supplement: Supplementary file 2 — Supplementary Information [file 42003_2021_2628_MOESM2_ESM.pdf]

Platinized graphene fiber electrodes uncover direct spleen-vagus communication

Maria A. Gonzalez-Gonzalez[1], Geetanjali S. Bendale[1,], Kezhong Wang[2], Gordon G. Wallace [2],

Mario Romero-Ortega*[1,]

Supplementary Information

* Corresponding author information:
Mario Romero-Ortega, PhD.
Biomedical Engineering and Biomedical Sciences.
Health 2. 4849 Calhoun Rd. Room 6014.
University of Houston. Houston TX. 77204-6064
Email: miromeroortega@uh.edu

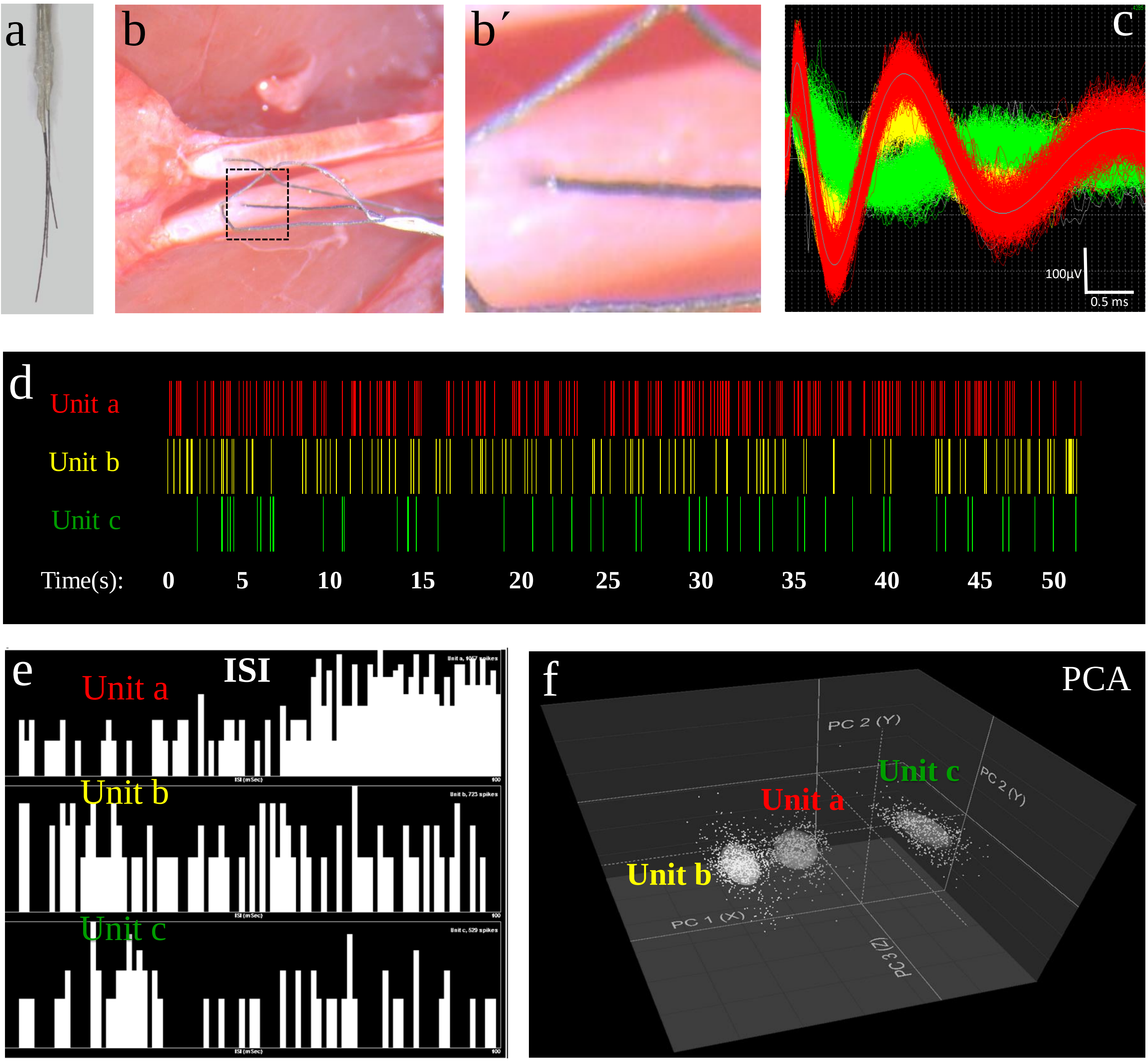

Supplementary Figure 1. Sutrode multi-array as intraneural peripheral interface. a) Sutrode multi-array; b) sutrode multi-array implanted intraneural in two fascicles of the ScN, magnification is displayed in b´. c) representative recording from one of the electrodes identifies three single units. Raster plots, inter-spike interval analysis (ISI), and principal component analysis (PCA) are presented in d-f, respectively. SNR was calculated as 9.6.

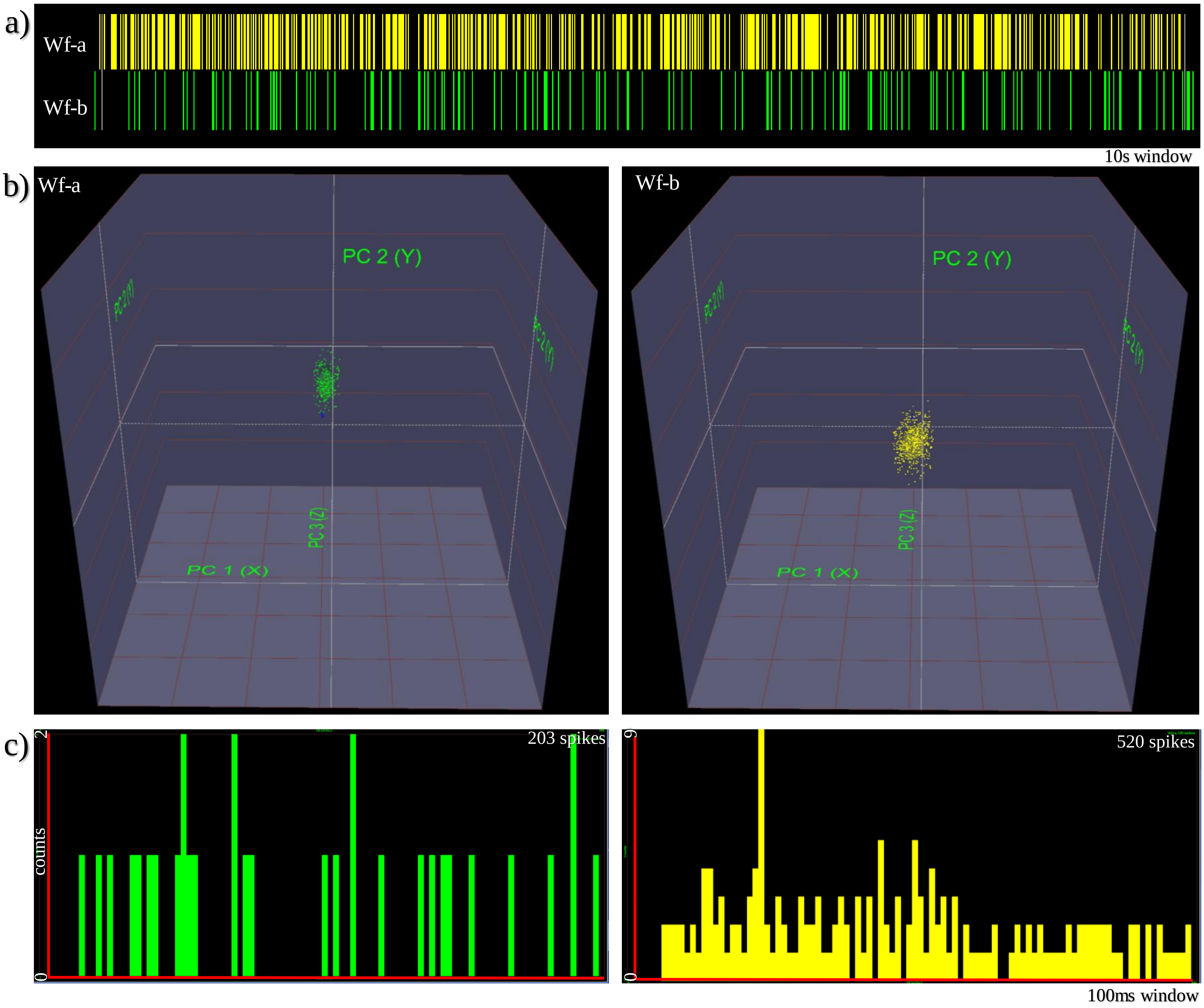

Supplementary Figure 2. Cervical vagus nerve (cVN) spontaneous activity measured with a sutrode. a) raster plots from two waveforms (Wf-a and b). Principal component analysis (PCA) and inter-spike interval analysis (ISI) for these units are presented in b and c, respectively.

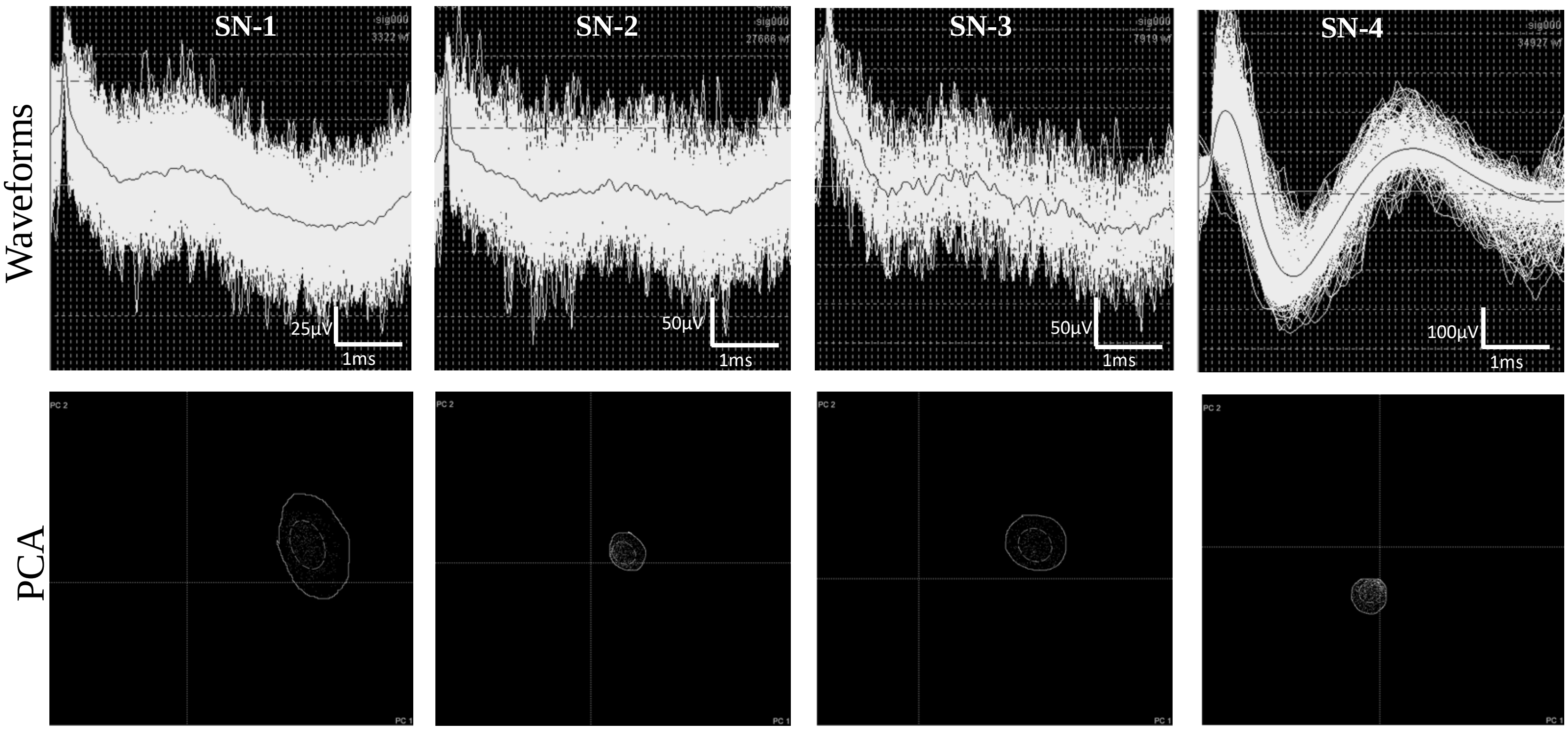

Supplementary Figure 3. Waveforms and principal component analysis (PCA) of splenic activity in response to hypoxia. Splenic neurovascular plexus in the spleen (SN-1 to 4) show waveforms identified by PCA.

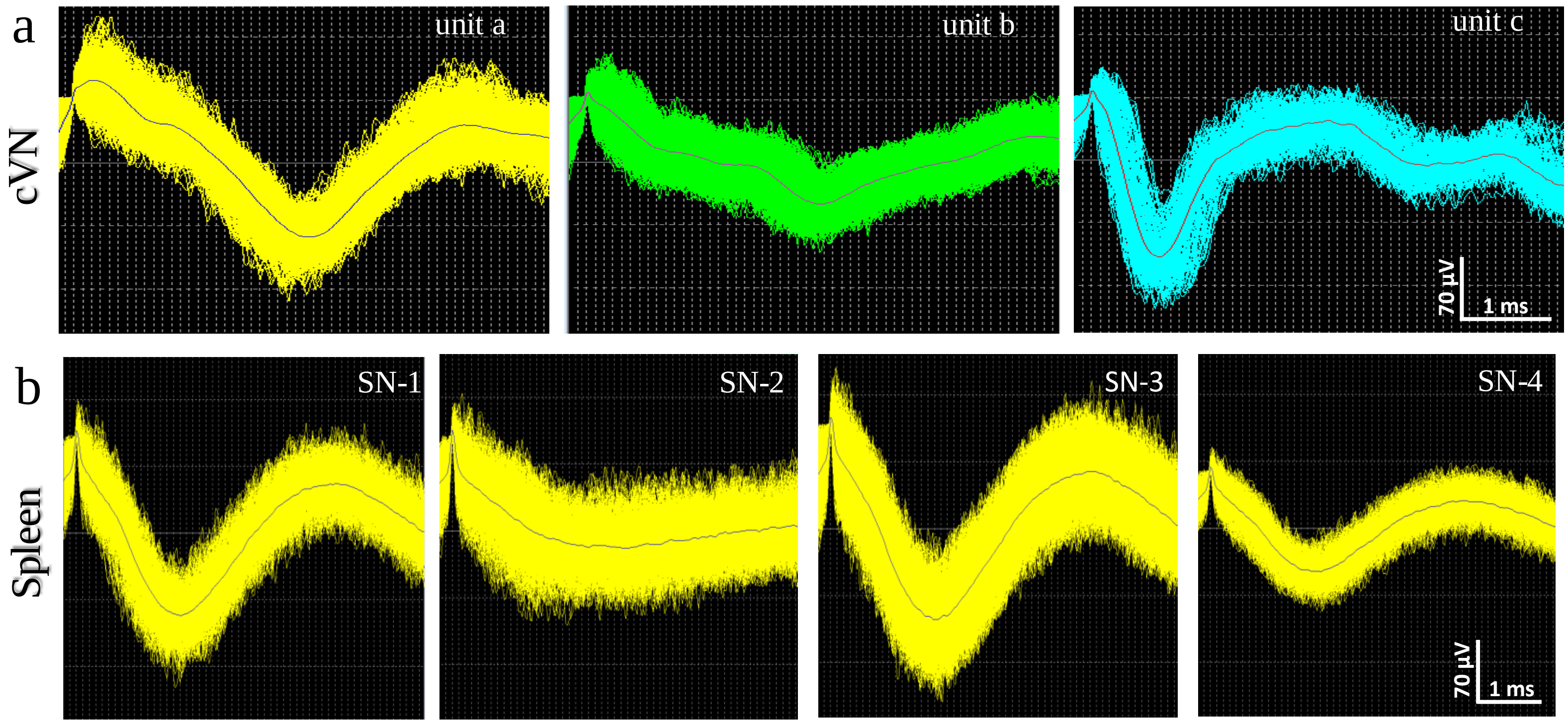

Delta of amplitude of signal
(baseline vs. NPS administration)

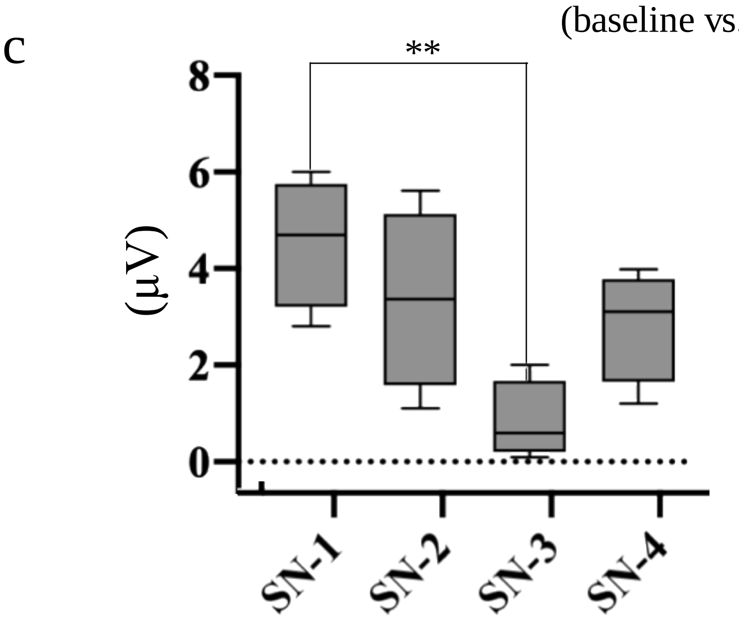

Targets	P value
SN-1 vs. SN-2	0.6783
SN-1 vs. SN-3**	0.0061
SN-1 vs.SN-4	0.3622
SN-2 vs. SN-3	0.0826
SN-2 vs. SN-4	0.9791
SN-3 vs. SN-4	0.2162

Alpha=0.05

Supplementary Figure 4. Cervical vagus nerve (cVN) and splenic response to mean arterial pressure decrease. Waveforms recorded from the cVN (a) and four splenic neurovascular plexus (SN1-4) in response to blood pressure drop, evoked by systemic administration of the vasoactive drug nitroprusside. Scale bars for unit c and SN-4 correspond to panels in (a) and (b), respectively. c) Amplitude (µV) changes in SN-1 to 4 and cVN at 200s after nitroprusside administration (compared to baseline). In the left is displayed a graphic with means and standard deviation values (n=5 animals, SN-1 to 4: 4.6±1.3, 3.4±1.9, 0.9±0.8, 2.8±1.2, respectively) and in the right, P values (alpha=0.05) for SN1-4, obtained from a Tukey's multiple comparisons test.

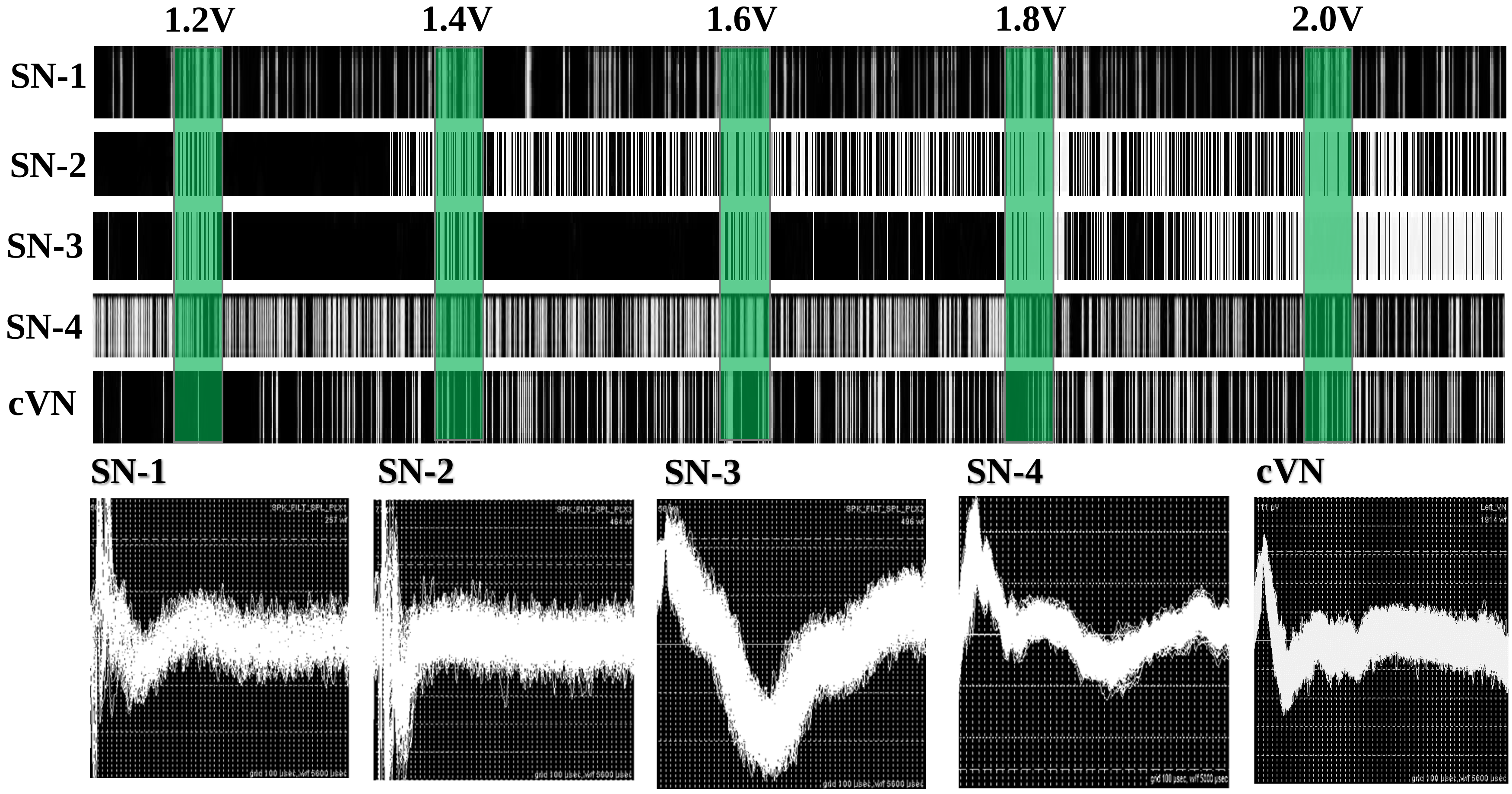

Supplementary Figure 5. cVN stimulation evokes voltaje-dependent activity in SN terminal branches. Neural activity in SN-1 to 4 and cVN, evoked by cVN stimulation: 0.3 ms biphasic pulses, at 2 Hz, during 30 s at 1.2, 1.4, 1.6, 1.8 and 2.0V.

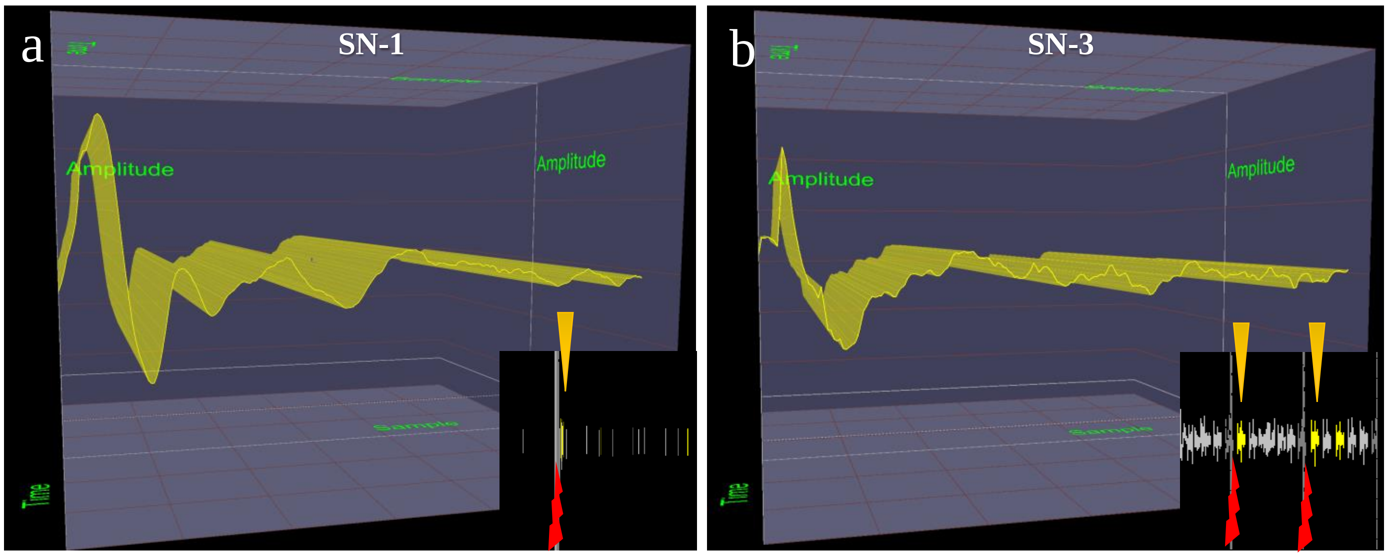

Supplementary Figure 6. Splenic response to subdiaphragmatic vagus nerve stimulation (SDVNs). a-b) Neuronal activity in splenic neurovascular plexus 1 and 3 (SN-1 and 3) is observed in a range of 1-3 ms (yellow arrowheads) after the stimulus artifact due to SDVNs (red symbols). The waveform for each SN is presented. X-axes segments are 1 ms, and in Y 10 μV.

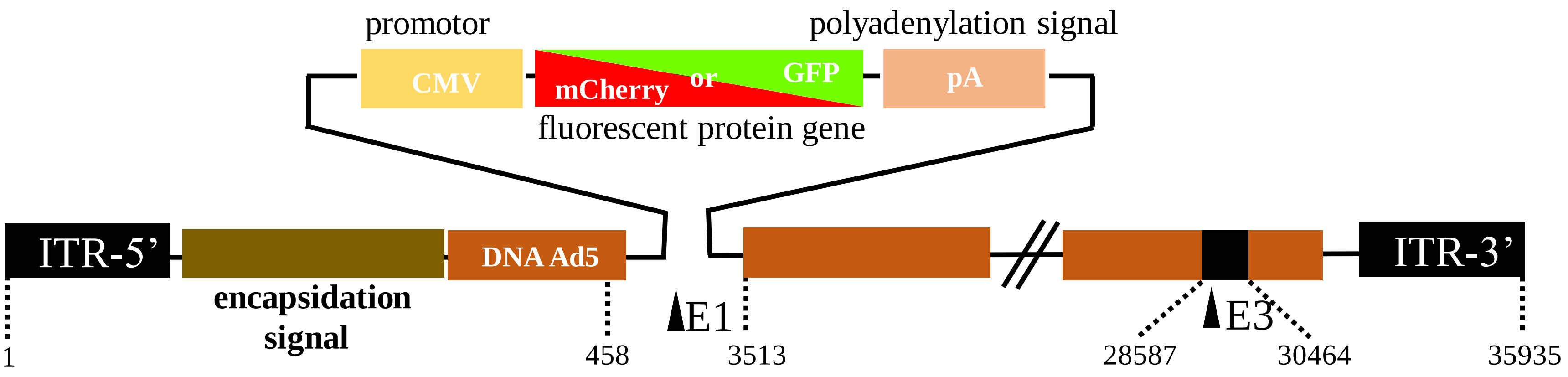

Supplementary Figure 7. Schematic representation of AdGFP and AdmCherry genome. The genes codifying for GFP or mCherry are cloned in the adenovirus type 5 (Ad5) and use as transcription promotor the human cytomegalovirus sequence (CMV). E1 and E3, early transcription genes; ITR, inverted terminal repeats.

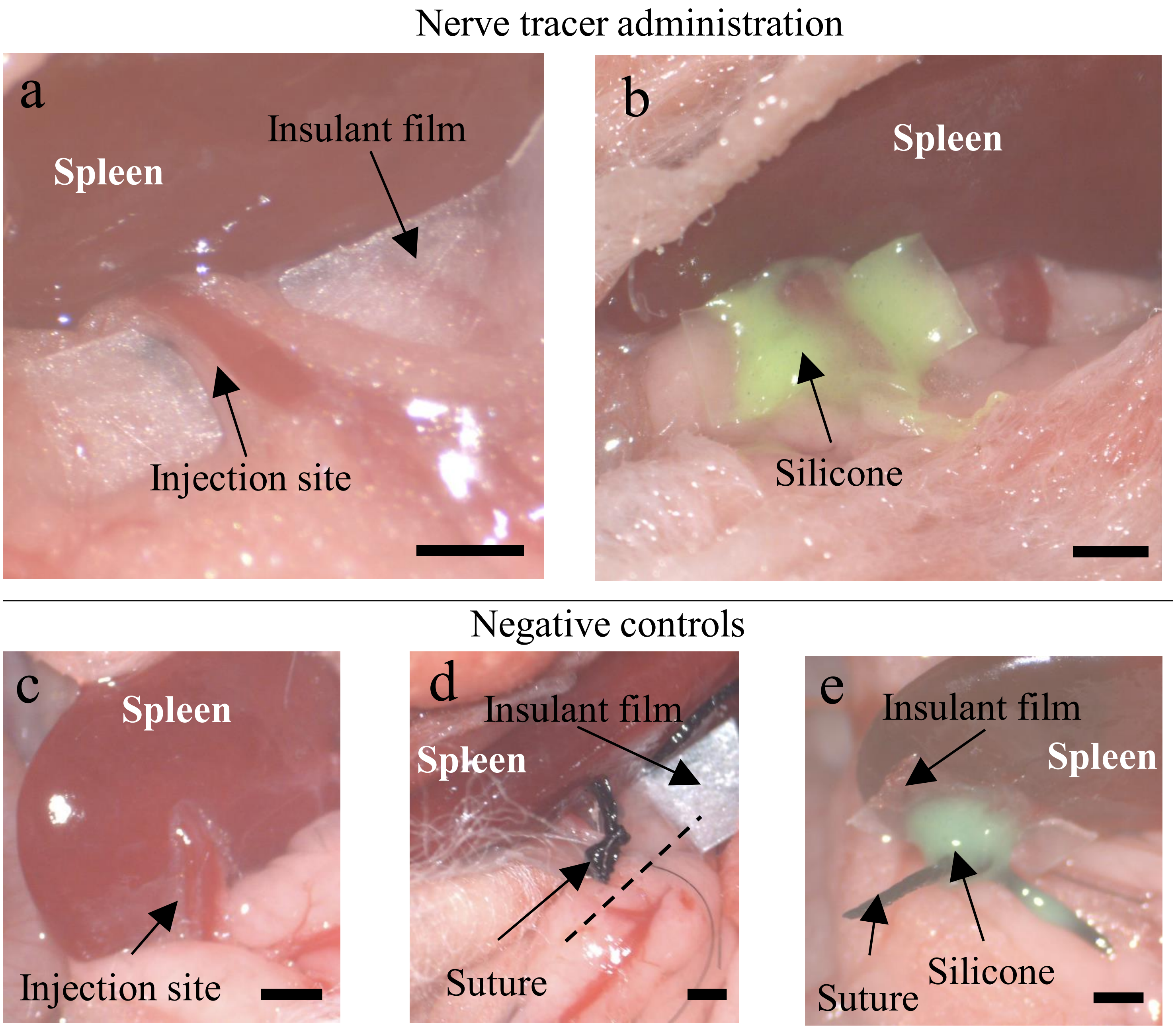

Supplementary Figure 8. Nerve tract-tracer administration. The viral tracer (Ad-GFP) was administrated in the splenic terminal neurovascular plexus (a and c), an insulant film was used to prevent leaking during the administration, followed by the application of biocompatible silicone to reinforce the prevention of tracer spreading (b and e). The negative controls consisted of transecting the splenic branch (dotted line) and adding a suture to prevent bleeding (d and e). Bar = 1mm.

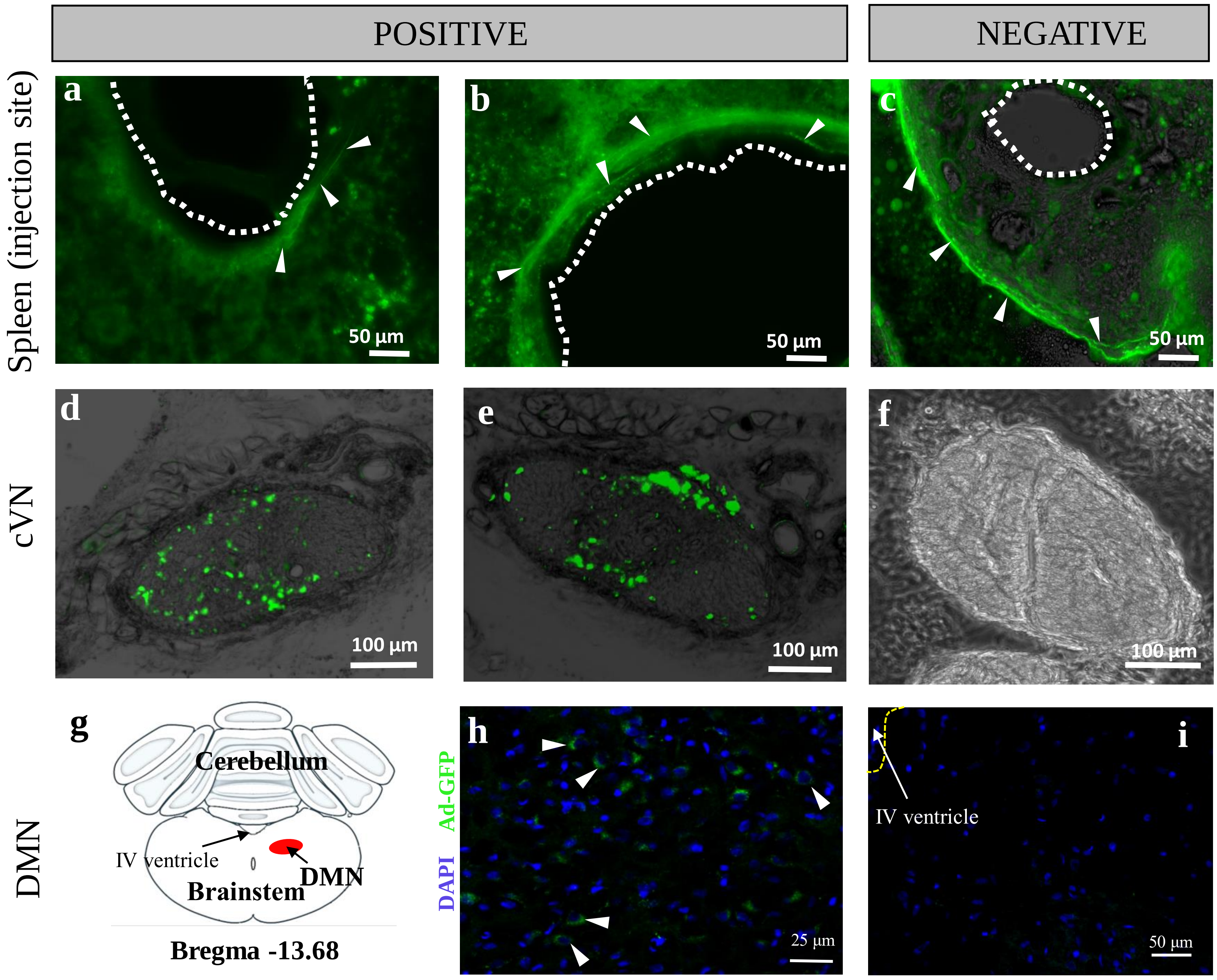

Supplementary Figure 9. Spleen – DMN adenoviral tract tracing. a-b) Images of two of seven positive rats administrated with Ad-GFP in the spleen, where nerve fibers were transduced (arrowheads) around the blood vessel forming the neuro-vascular plexus (dotted lines). d-e) Cross-sections of the cVN with GFP+ axons (green signal). Negative controls consisted of transecting the SNVP after viral administration, images from the spleen and cVN are represented in c and f. h-i) Positive and negative images of a coronal section of the brain (as shown in g) including the DMN. (n=7 positives, n=5 negative controls). DMN, dorsal motor nucleus of the vagus; cVN, cervical vagus nerve; SNVP, splenic neuro-vascular plexus; GFP, green fluorescent protein.

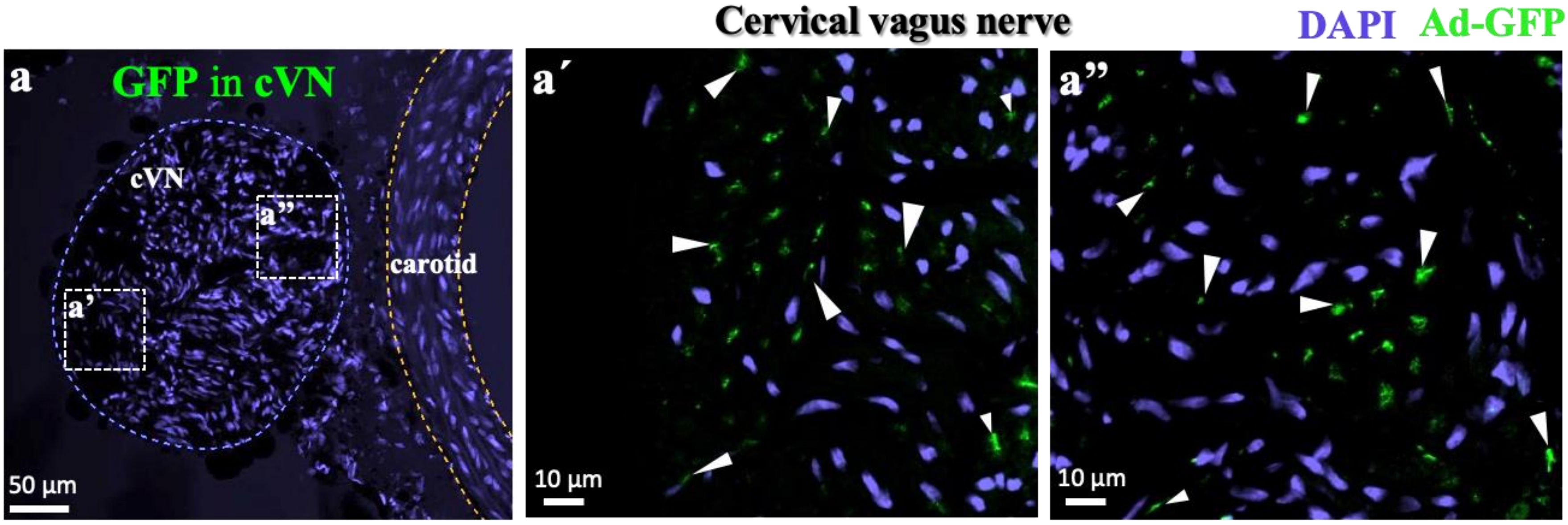

Supplementary Figure 10. AdGFP+ clustered axons in the cVN. a) Cross-section of representative cVN, adjacent to the carotid artery (blue and yellow dotted lines); magnifications presented as a' and a'' show two GFP+ axonal clusters. Cellular nuclei were stained with DAPI. cVN, cervical vagus nerve; GFP, green fluorescent protein; AdGFP, adenoviral signal transduced as GFP.
