# Supplementary material for: Platinized graphene fiber electrodes uncover direct spleen-vagus communication
Source: Commun Biol. 2021 Sep 17;4:1097. doi: 10.1038/s42003-021-02628-7 (PMC8448843; doi:10.1038/s42003-021-02628-7)
Supplement: Supplementary file 3 — Description of Supplementary Files [file 42003_2021_2628_MOESM3_ESM.pdf]

Description of Additional Supplementary Files

File name: Supplementary Video 1

Description: The sutrode flexibility and impedance stability when making a knot.

File name: Supplementary Video 2

Description: The sutrode is used as conductive suture for muscle stimulation.

File name: Supplementary Video 3

Description: Splenic neurovascular plexus terminal branches.

File name: Supplementary Data 1

Description: Data underlying graphs and charts.
