# Supplementary material for: Platinized graphene fiber electrodes uncover direct spleen-vagus communication
Source: Commun Biol. 2021 Sep 17;4:1097. doi: 10.1038/s42003-021-02628-7 (PMC8448843; doi:10.1038/s42003-021-02628-7)
Supplement: Supplementary file 8 — Reporting Summary [file 42003_2021_2628_MOESM8_ESM.pdf]

nature research

Corresponding author(s): DBPR COMMSBIO-20-1911B

Last updated by author(s): 8/6/21 - Mario Romero-Ortega

Reporting Summary

Nature Research wishes to improve the reproducibility of the work that we publish. This form provides structure for consistency and transparency in reporting. For further information on Nature Research policies, see our Editorial Policies and the Editorial Policy Checklist.

Statistics

For all statistical analyses, confirm that the following items are present in the figure legend, table legend, main text, or Methods section.

n/a	Confirmed	
☐	☒	The exact sample size (*n*) for each experimental group/condition, given as a discrete number and unit of measurement
☐	☒	A statement on whether measurements were taken from distinct samples or whether the same sample was measured repeatedly
☐	☒	The statistical test(s) used AND whether they are one- or two-sided<br>*Only common tests should be described solely by name; describe more complex techniques in the Methods section.*
☐	☒	A description of all covariates tested
☐	☒	A description of any assumptions or corrections, such as tests of normality and adjustment for multiple comparisons
☐	☒	A full description of the statistical parameters including central tendency (e.g. means) or other basic estimates (e.g. regression coefficient) AND variation (e.g. standard deviation) or associated estimates of uncertainty (e.g. confidence intervals)
☐	☒	For null hypothesis testing, the test statistic (e.g. *F*, *t*, *r*) with confidence intervals, effect sizes, degrees of freedom and *P* value noted<br>*Give P values as exact values whenever suitable.*
☒	☐	For Bayesian analysis, information on the choice of priors and Markov chain Monte Carlo settings
☒	☐	For hierarchical and complex designs, identification of the appropriate level for tests and full reporting of outcomes
☒	☐	Estimates of effect sizes (e.g. Cohen's *d*, Pearson's *r*), indicating how they were calculated

Our web collection on statistics for biologists contains articles on many of the points above.

Software and code

Policy information about availability of computer code

Data collection	Omniplex data acquisition system (Plexon Inc. Dallas TX) was used for electrophysiological data collection. A PlexStim stimulator system (Plexon Inc) was used for nerves electrical stimulation. A pressure transducer (AD-Instruments, MLT1199) coupled to a bridge amplifier and power supply modulus (AD-Instruments, FE221 and ML826, respectively) were used for mean arterial pressure measurements. A PowerLab data acquisition system software (AD Instruments, Colorado Springs, CO) was used to aquire the mean arterial pressure. A scanning electron microscope JEOL JSM-7500FA was used to image the sutrodes. A transmission electron microscope (TEM, JEOL 1400 Plus, JEOL, USA) was used for nerve ultrastructure analysis. A confocal microscope (Nikon Eclipse Ti, Japan) was used to visualize fluorescent signal on the nerve tract tracing studies. A digital multimeter (Agilent 34401A) was used to record the impedance of the sutrodes.
Data analysis	Off-line sorter software (Plexon Inc, v3.3.5) and NeuroExplorer software (Nex Technologies, v 4.135) were used for electrophysiological data analysis. LabChart Pro V8 (AD Instruments, Colorado Springs, CO) was used to process and analyze the mean arterial pressure data. FIJI ImageJ was used for image analysis (Schindelin, J.; Arganda-Carreras, I. & Frise, E. et al. (2012), "Fiji: an open-source platform for biological-image analysis", Nature methods 9(7): 676-682, PMID 22743772, doi:10.1038/nmeth.2019).

For manuscripts utilizing custom algorithms or software that are central to the research but not yet described in published literature, software must be made available to editors and reviewers. We strongly encourage code deposition in a community repository (e.g. GitHub). See the Nature Research guidelines for submitting code & software for further information.

Data

Policy information about availability of data

All manuscripts must include a data availability statement. This statement should provide the following information, where applicable:

- Accession codes, unique identifiers, or web links for publicly available datasets
- A list of figures that have associated raw data
- A description of any restrictions on data availability

All relevant data are included in the paper. The data used to prepare the figures are provided in an excel document as supplementary information.

Field-specific reporting

Please select the one below that is the best fit for your research. If you are not sure, read the appropriate sections before making your selection.

☒ Life sciences ☐ Behavioural & social sciences ☐ Ecological, evolutionary & environmental sciences

For a reference copy of the document with all sections, see nature.com/documents/nr-reporting-summary-flat.pdf

Life sciences study design

All studies must disclose on these points even when the disclosure is negative.

Sample size	The number of animals used in this study and total number of test are indicated.
Data exclusions	Data collected from rats that went through long periods of anesthesia (i.e. >3h) was excluded to prevent physiological artifacts.
Replication	The data obtained in this study was replicable between animals, and statistical significance was demonstrated.
Randomization	In the studies where different electrical parameters of stimulation where used, a randomization strategy was applied. For the studies on pharmacological modulation of blood pressure, periods of time (>30 min) were allowed for the system to return to basal conditions.
Blinding	The experimental design for this study did not require a data blinding stage.

Reporting for specific materials, systems and methods

We require information from authors about some types of materials, experimental systems and methods used in many studies. Here, indicate whether each material, system or method listed is relevant to your study. If you are not sure if a list item applies to your research, read the appropriate section before selecting a response.

Materials & experimental systems

n/a	Involved in the study	
☒	☐	Antibodies
☒	☐	Eukaryotic cell lines
☒	☐	Palaeontology and archaeology
☐	☒	Animals and other organisms
☒	☐	Human research participants
☒	☐	Clinical data
☒	☐	Dual use research of concern

Methods

n/a	Involved in the study	
☒	☐	ChIP-seq
☒	☐	Flow cytometry
☒	☐	MRI-based neuroimaging

Animals and other organisms

Policy information about studies involving animals; ARRIVE guidelines recommended for reporting animal research

Laboratory animals	Female Sprague Dawley rats (300-350 g; Charles River, Wilmington, MA) were used for this study.
Wild animals	The study did not involve wild animals
Field-collected samples	The study did not involve field-collected samples.
Ethics oversight	Animal protocols for this study were approved by The University of Texas at Dallas, Institutional Animal Care and Use Committee (IACUC, protocol No.14-09), and at the University of Houston (IACUC, protocol 202000004).

Note that full information on the approval of the study protocol must also be provided in the manuscript.
